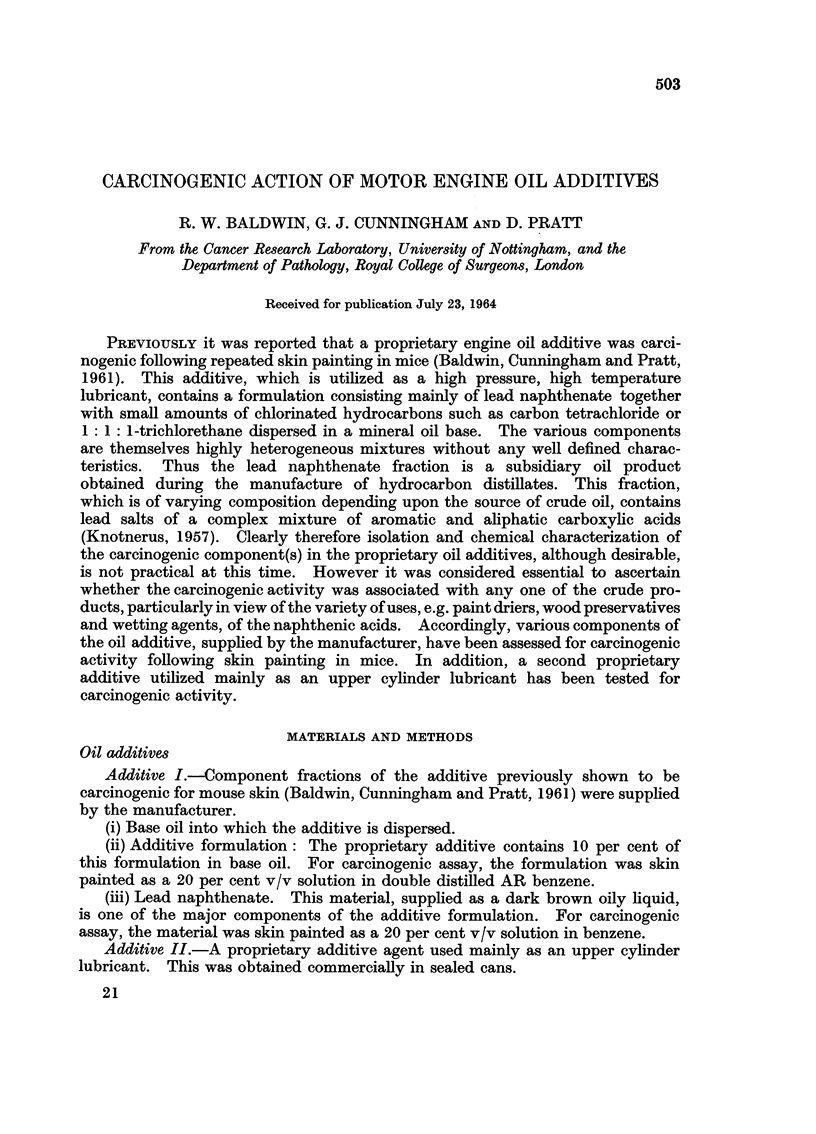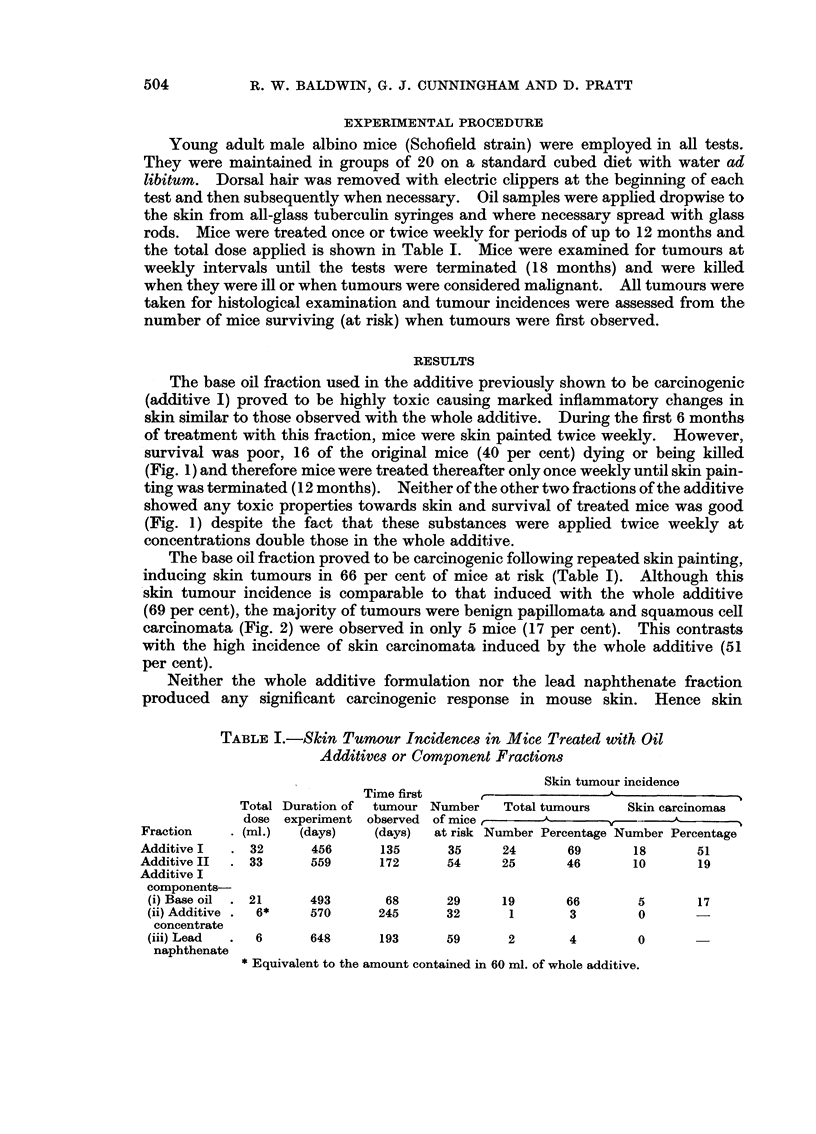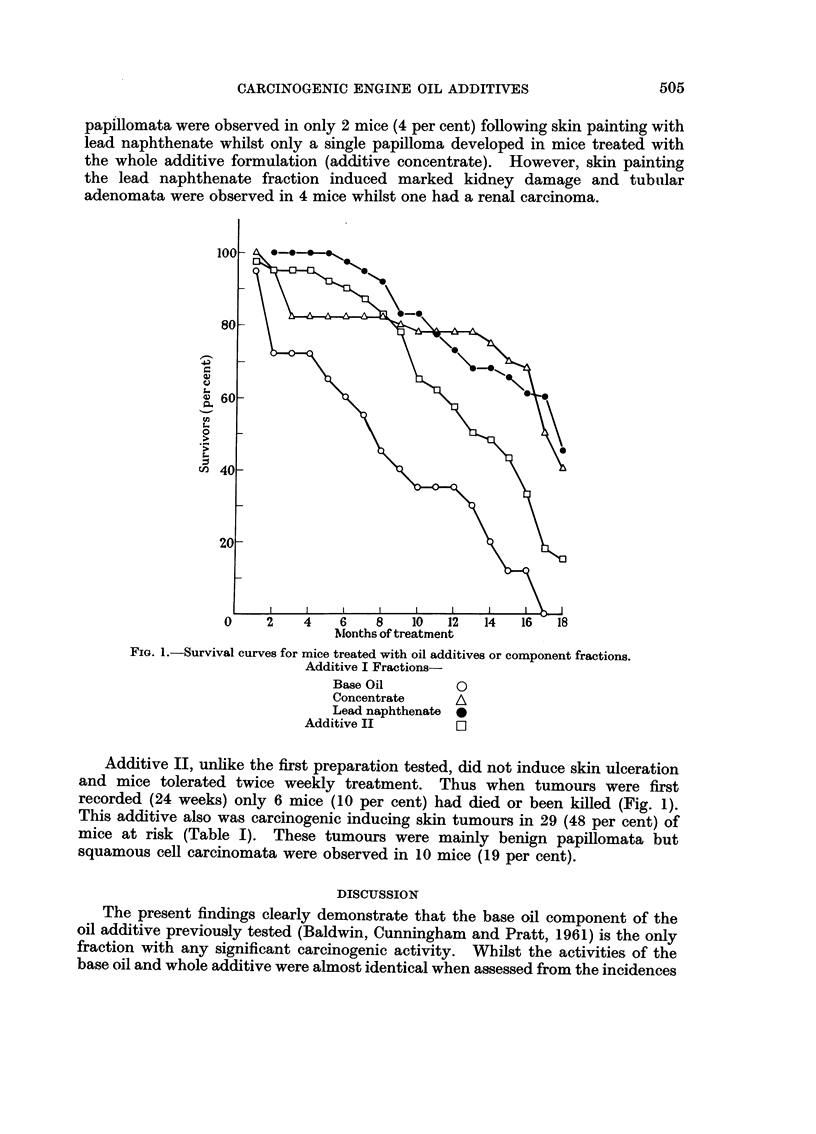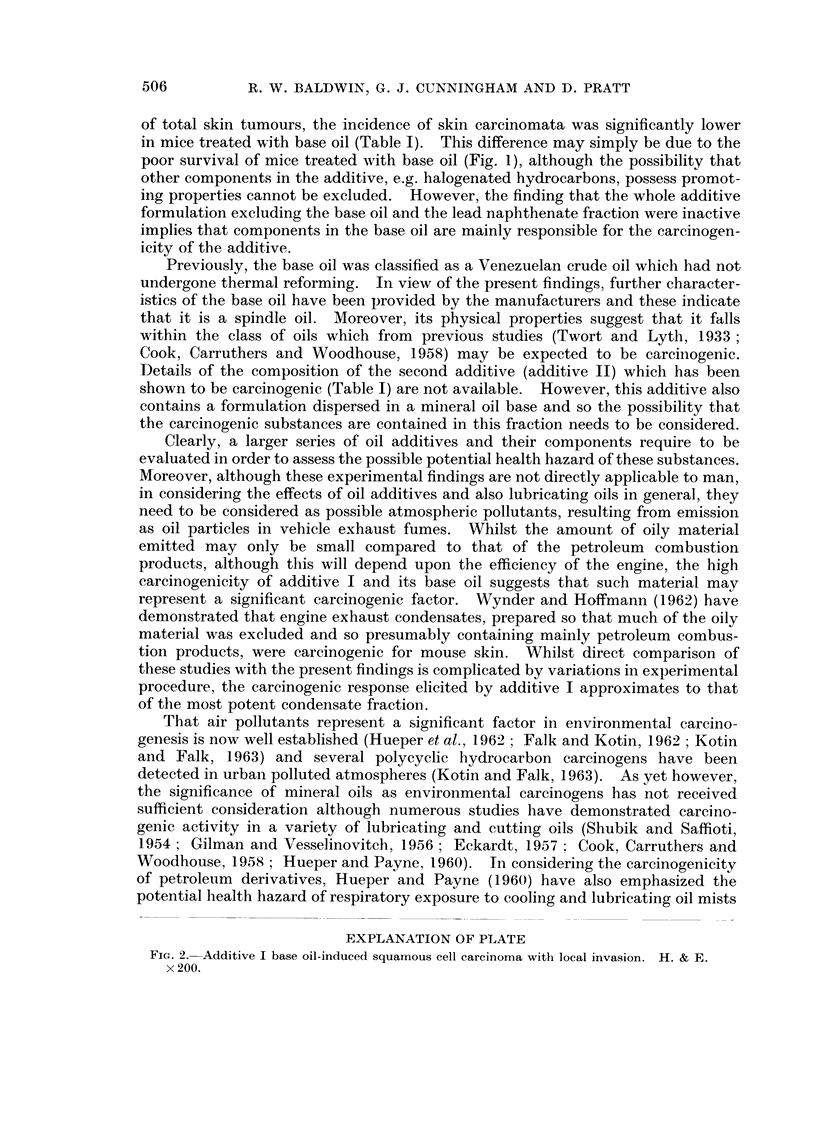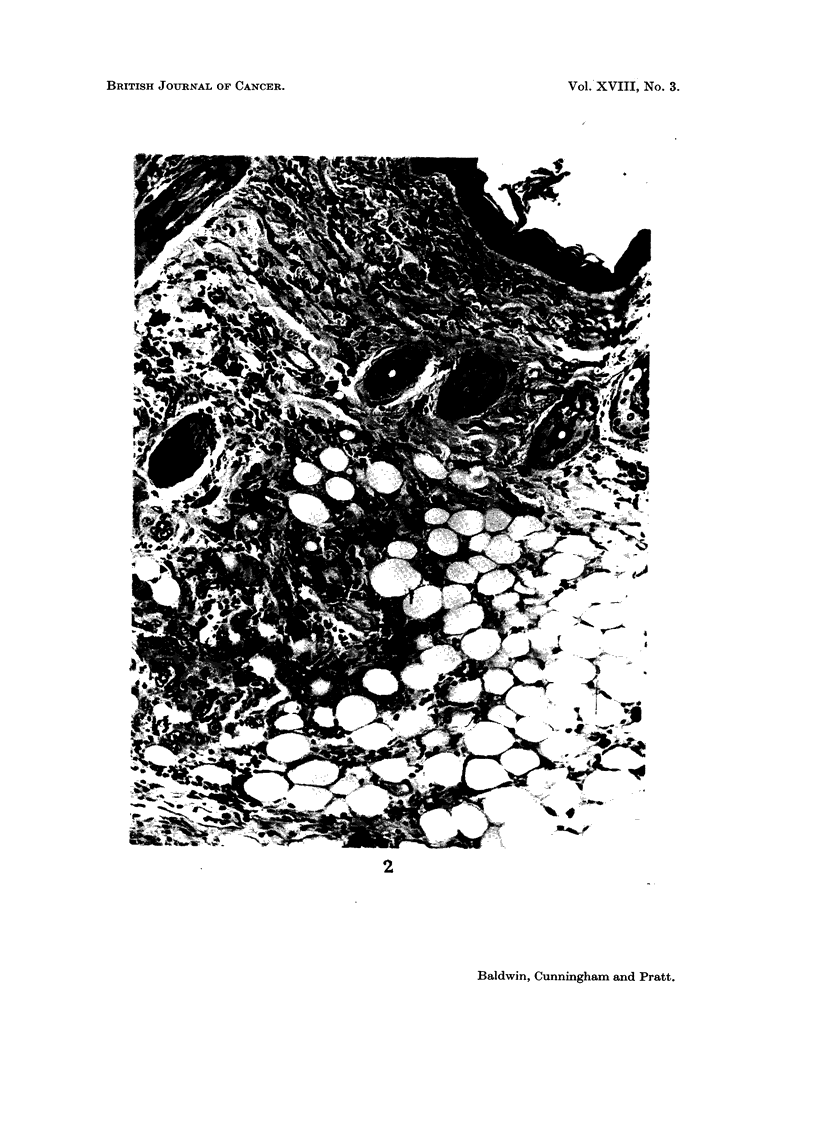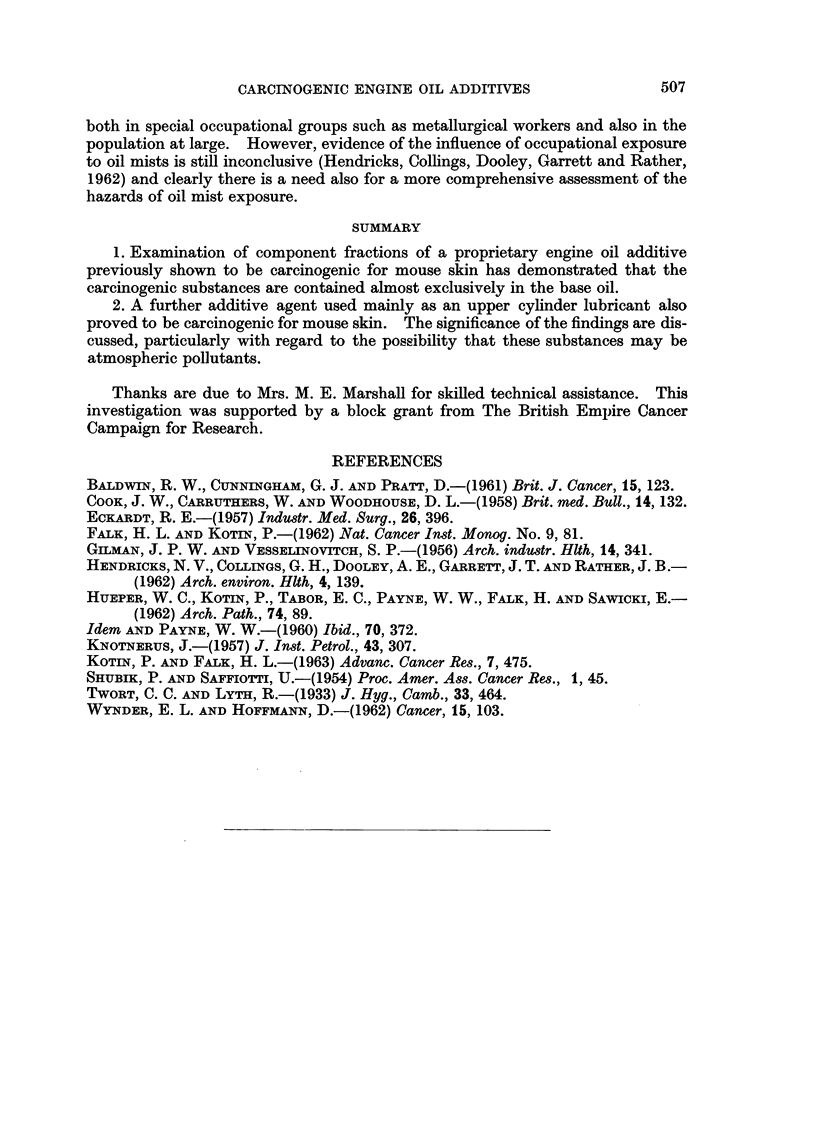# Carcinogenic Action of Motor Engine Oil Additives

**DOI:** 10.1038/bjc.1964.56

**Published:** 1964-09

**Authors:** R. W. Baldwin, G. J. Cunningham, D. Pratt

## Abstract

**Images:**


### CARCINOGENIC ACTION OF MOTOR ENGINE OIL ADDITIVES

R. W. BALDWIN, G. J. CUNNINGHAM AND D. PRATT

*From the Cancer Research Laboratory, University of Nottingham, and the Department of Pathology, Royal College of Surgeons, London*

Received for publication July 23, 1964

PREVIOUSLY it was reported that a proprietary engine oil additive was carcinogenic following repeated skin painting in mice (Baldwin, Cunningham and Pratt, 1961). This additive, which is utilized as a high pressure, high temperature lubricant, contains a formulation consisting mainly of lead naphthenate together with small amounts of chlorinated hydrocarbons such as carbon tetrachloride or 1 : 1 : 1-trichlorethane dispersed in a mineral oil base. The various components are themselves highly heterogeneous mixtures without any well defined characteristics. Thus the lead naphthenate fraction is a subsidiary oil product obtained during the manufacture of hydrocarbon distillates. This fraction, which is of varying composition depending upon the source of crude oil, contains lead salts of a complex mixture of aromatic and aliphatic carboxylic acids (Knotnerus, 1957). Clearly therefore isolation and chemical characterization of the carcinogenic component(s) in the proprietary oil additives, although desirable, is not practical at this time. However it was considered essential to ascertain whether the carcinogenic activity was associated with any one of the crude products, particularly in view of the variety of uses, e.g. paint driers, wood preservatives and wetting agents, of the naphthenic acids. Accordingly, various components of the oil additive, supplied by the manufacturer, have been assessed for carcinogenic activity following skin painting in mice. In addition, a second proprietary additive utilized mainly as an upper cylinder lubricant has been tested for carcinogenic activity.

### MATERIALS AND METHODS

### *Oil additives*

*Additive I*.—Component fractions of the additive previously shown to be carcinogenic for mouse skin (Baldwin, Cunningham and Pratt, 1961) were supplied by the manufacturer.

(i) Base oil into which the additive is dispersed.

(ii) Additive formulation : The proprietary additive contains 10 per cent of this formulation in base oil. For carcinogenic assay, the formulation was skin painted as a 20 per cent v/v solution in double distilled AR benzene.

(iii) Lead naphthenate. This material, supplied as a dark brown oily liquid, is one of the major components of the additive formulation. For carcinogenic assay, the material was skin painted as a 20 per cent v/v solution in benzene.

*Additive II*.—A proprietary additive agent used mainly as an upper cylinder lubricant. This was obtained commercially in sealed cans.

### EXPERIMENTAL PROCEDURE

Young adult male albino mice (Schofield strain) were employed in all tests. They were maintained in groups of 20 on a standard cubed diet with water *ad libitum*. Dorsal hair was removed with electric clippers at the beginning of each test and then subsequently when necessary. Oil samples were applied dropwise to the skin from all-glass tuberculin syringes and where necessary spread with glass rods. Mice were treated once or twice weekly for periods of up to 12 months and the total dose applied is shown in Table I. Mice were examined for tumours at weekly intervals until the tests were terminated (18 months) and were killed when they were ill or when tumours were considered malignant. All tumours were taken for histological examination and tumour incidences were assessed from the number of mice surviving (at risk) when tumours were first observed.

### RESULTS

The base oil fraction used in the additive previously shown to be carcinogenic (additive I) proved to be highly toxic causing marked inflammatory changes in skin similar to those observed with the whole additive. During the first 6 months of treatment with this fraction, mice were skin painted twice weekly. However, survival was poor, 16 of the original mice (40 per cent) dying or being killed (Fig. 1) and therefore mice were treated thereafter only once weekly until skin painting was terminated (12 months). Neither of the other two fractions of the additive showed any toxic properties towards skin and survival of treated mice was good (Fig. 1) despite the fact that these substances were applied twice weekly at concentrations double those in the whole additive.

The base oil fraction proved to be carcinogenic following repeated skin painting, inducing skin tumours in 66 per cent of mice at risk (Table I). Although this skin tumour incidence is comparable to that induced with the whole additive (69 per cent), the majority of tumours were benign papillomata and squamous cell carcinomata (Fig. 2) were observed in only 5 mice (17 per cent). This contrasts with the high incidence of skin carcinomata induced by the whole additive (51 per cent).

Neither the whole additive formulation nor the lead naphthenate fraction produced any significant carcinogenic response in mouse skin. Hence skin

TABLE I.—*Skin Tumour Incidences in Mice Treated with Oil Additives or Component Fractions*

| | | | | | Skin tumour incidence | | | |
|---|---|---|---|---|---|---|---|---|
| | | | | | Total tumours | | Skin carcinomas | |
| Fraction | Total dose (ml.) | Duration of experiment (days) | Time first tumour observed (days) | Number of mice at risk | Number | Percentage | Number | Percentage |
| Additive I | 32 | 456 | 135 | 35 | 24 | 69 | 18 | 51 |
| Additive II | 33 | 559 | 172 | 54 | 25 | 46 | 10 | 19 |
| Additive I components— | | | | | | | | |
| (i) Base oil | 21 | 493 | 68 | 29 | 19 | 66 | 5 | 17 |
| (ii) Additive concentrate | 6* | 570 | 245 | 32 | 1 | 3 | 0 | — |
| (iii) Lead naphthenate | 6 | 648 | 193 | 59 | 2 | 4 | 0 | — |

* Equivalent to the amount contained in 60 ml. of whole additive.

papillomata were observed in only 2 mice (4 per cent) following skin painting with lead naphthenate whilst only a single papilloma developed in mice treated with the whole additive formulation (additive concentrate). However, skin painting the lead naphthenate fraction induced marked kidney damage and tubular adenomata were observed in 4 mice whilst one had a renal carcinoma.

FIG. 1.—Survival curves for mice treated with oil additives or component fractions.
Additive I Fractions—
Base Oil ○
Concentrate △
Lead naphthenate ●
Additive II □

Additive II, unlike the first preparation tested, did not induce skin ulceration and mice tolerated twice weekly treatment. Thus when tumours were first recorded (24 weeks) only 6 mice (10 per cent) had died or been killed (Fig. 1). This additive also was carcinogenic inducing skin tumours in 29 (48 per cent) of mice at risk (Table I). These tumours were mainly benign papillomata but squamous cell carcinomata were observed in 10 mice (19 per cent).

### DISCUSSION

The present findings clearly demonstrate that the base oil component of the oil additive previously tested (Baldwin, Cunningham and Pratt, 1961) is the only fraction with any significant carcinogenic activity. Whilst the activities of the base oil and whole additive were almost identical when assessed from the incidences of total skin tumours, the incidence of skin carcinomata was significantly lower in mice treated with base oil (Table I). This difference may simply be due to the poor survival of mice treated with base oil (Fig. 1), although the possibility that other components in the additive, e.g. halogenated hydrocarbons, possess promoting properties cannot be excluded. However, the finding that the whole additive formulation excluding the base oil and the lead naphthenate fraction were inactive implies that components in the base oil are mainly responsible for the carcinogenicity of the additive.

Previously, the base oil was classified as a Venezuelan crude oil which had not undergone thermal reforming. In view of the present findings, further characteristics of the base oil have been provided by the manufacturers and these indicate that it is a spindle oil. Moreover, its physical properties suggest that it falls within the class of oils which from previous studies (Twort and Lyth, 1933 ; Cook, Carruthers and Woodhouse, 1958) may be expected to be carcinogenic. Details of the composition of the second additive (additive II) which has been shown to be carcinogenic (Table I) are not available. However, this additive also contains a formulation dispersed in a mineral oil base and so the possibility that the carcinogenic substances are contained in this fraction needs to be considered.

Clearly, a larger series of oil additives and their components require to be evaluated in order to assess the possible potential health hazard of these substances. Moreover, although these experimental findings are not directly applicable to man, in considering the effects of oil additives and also lubricating oils in general, they need to be considered as possible atmospheric pollutants, resulting from emission as oil particles in vehicle exhaust fumes. Whilst the amount of oily material emitted may only be small compared to that of the petroleum combustion products, although this will depend upon the efficiency of the engine, the high carcinogenicity of additive I and its base oil suggests that such material may represent a significant carcinogenic factor. Wynder and Hoffmann (1962) have demonstrated that engine exhaust condensates, prepared so that much of the oily material was excluded and so presumably containing mainly petroleum combustion products, were carcinogenic for mouse skin. Whilst direct comparison of these studies with the present findings is complicated by variations in experimental procedure, the carcinogenic response elicited by additive I approximates to that of the most potent condensate fraction.

That air pollutants represent a significant factor in environmental carcinogenesis is now well established (Hueper *et al.*, 1962 ; Falk and Kotin, 1962 ; Kotin and Falk, 1963) and several polycyclic hydrocarbon carcinogens have been detected in urban polluted atmospheres (Kotin and Falk, 1963). As yet however, the significance of mineral oils as environmental carcinogens has not received sufficient consideration although numerous studies have demonstrated carcinogenic activity in a variety of lubricating and cutting oils (Shubik and Saffioti, 1954 ; Gilman and Vesselinovitch, 1956 ; Eckardt, 1957 ; Cook, Carruthers and Woodhouse, 1958 ; Hueper and Payne, 1960). In considering the carcinogenicity of petroleum derivatives, Hueper and Payne (1960) have also emphasized the potential health hazard of respiratory exposure to cooling and lubricating oil mists

EXPLANATION OF PLATE

FIG. 2.—Additive I base oil-induced squamous cell carcinoma with local invasion. H. & E. ×200.

2

Baldwin, Cunningham and Pratt.

both in special occupational groups such as metallurgical workers and also in the population at large. However, evidence of the influence of occupational exposure to oil mists is still inconclusive (Hendricks, Collings, Dooley, Garrett and Rather, 1962) and clearly there is a need also for a more comprehensive assessment of the hazards of oil mist exposure.

SUMMARY

1. Examination of component fractions of a proprietary engine oil additive previously shown to be carcinogenic for mouse skin has demonstrated that the carcinogenic substances are contained almost exclusively in the base oil.

2. A further additive agent used mainly as an upper cylinder lubricant also proved to be carcinogenic for mouse skin. The significance of the findings are discussed, particularly with regard to the possibility that these substances may be atmospheric pollutants.

Thanks are due to Mrs. M. E. Marshall for skilled technical assistance. This investigation was supported by a block grant from The British Empire Cancer Campaign for Research.

### REFERENCES

Baldwin, R. W., Cunningham, G. J. and Pratt, D.—(1961) *Brit. J. Cancer*, **15**, 123.
Cook, J. W., Carruthers, W. and Woodhouse, D. L.—(1958) *Brit. med. Bull.*, **14**, 132.
Eckardt, R. E.—(1957) *Industr. Med. Surg.*, **26**, 396.
Falk, H. L. and Kotin, P.—(1962) *Nat. Cancer Inst. Monog.* No. 9, 81.
Gilman, J. P. W. and Vesselinovitch, S. P.—(1956) *Arch. industr. Hlth*, **14**, 341.
Hendricks, N. V., Collings, G. H., Dooley, A. E., Garrett, J. T. and Rather, J. B.—(1962) *Arch. environ. Hlth*, **4**, 139.
Hueper, W. C., Kotin, P., Tabor, E. C., Payne, W. W., Falk, H. and Sawicki, E.—(1962) *Arch. Path.*, **74**, 89.
*Idem* and Payne, W. W.—(1960) *Ibid.*, **70**, 372.
Knotnerus, J.—(1957) *J. Inst. Petrol.*, **43**, 307.
Kotin, P. and Falk, H. L.—(1963) *Advanc. Cancer Res.*, **7**, 475.
Shubik, P. and Saffiotti, U.—(1954) *Proc. Amer. Ass. Cancer Res.*, **1**, 45.
Twort, C. C. and Lyth, R.—(1933) *J. Hyg., Camb.*, **33**, 464.
Wynder, E. L. and Hoffmann, D.—(1962) *Cancer*, **15**, 103.